# Post-traumatic End-Stage Hip Osteoarthritis Decades After an Adolescent Gunshot Wound Treated With Direct Anterior Total Hip Arthroplasty: A Case Report

**DOI:** 10.7759/cureus.103899

**Published:** 2026-02-19

**Authors:** Amir Mirnateghi, Vivek Boddakayala, Konstantin M Nakov, Chelsea A Alton, Jeffrey Burnette

**Affiliations:** 1 Medicine, Lake Erie College of Osteopathic Medicine, Bradenton, USA; 2 Orthopedic Surgery, Southeast Orthopedic Specialists, Jacksonville, USA

**Keywords:** direct anterior approach, gunshot wound, leg length discrepancy, osteoarthritis, post-traumatic hip osteoarthritis, total hip arthroplasty: tha

## Abstract

Gunshot wounds to the pelvis during adolescence are uncommon but may result in long-term orthopedic complications, including pelvic asymmetry, acetabular deformity, and early development of hip osteoarthritis, which can complicate later reconstructive surgery. We describe the case of a 53-year-old woman who sustained a through-and-through gunshot injury to the left pelvis at age 16 and presented decades later with progressive left hip pain, functional limitation, and worsening leg length discrepancy. Imaging demonstrated severe post-traumatic end-stage osteoarthritis of the hip, acetabular deformity, pelvic obliquity, heterotopic ossification, and retained metallic fragments. After failure of nonoperative management, she underwent direct anterior total hip arthroplasty (THA) with preoperative CT-based planning and a strategy of partial limb-length correction to reduce neurologic risk. Postoperative radiographs confirmed appropriate implant positioning without evidence of loosening or subsidence. At two weeks postoperatively, the patient reported meaningful pain relief, improved mobility with a cane, and an uncomplicated wound healing course. This case illustrates that direct anterior THA can be successfully performed in the setting of complex post-traumatic deformity when meticulous preoperative planning and cautious intraoperative technique are employed.

## Introduction

Gunshot wounds to the pelvis during adolescence are rare but can cause long-term musculoskeletal problems, including growth arrest, pelvic asymmetry, acetabular deformity, and early-onset osteoarthritis [1,2]. These deformities make total hip arthroplasty (THA) technically difficult because of distorted anatomy, retained foreign bodies, heterotopic ossification, soft-tissue contractures, and an increased risk of nerve injury when correcting limb length [3-5].

We present a case of severe post-traumatic hip osteoarthritis that developed more than 30 years after a pelvic gunshot wound in adolescence and was treated successfully with direct anterior THA using CT-based preoperative planning [6,7].

## Case presentation

Patient information

A 53-year-old woman presented with chronic, worsening left hip pain. Her medical history included hypertension, mitral valve prolapse, thyroid disease, gastroesophageal reflux disease, arthritis, chronic knee pain, and a gunshot wound to the left hip and pelvis in 1988. She estimated she’d undergone about 27 surgeries related to her injury, including several growth-arrest procedures in an attempt to equalize her leg lengths.

She shared that she smokes about a pack of cigarettes each week and drinks alcohol only occasionally. She denied any illicit drug use. She has a BMI of 24.5 and is postmenopausal. Her medications included amlodipine, lisinopril, levothyroxine, omeprazole, and intermittent lorazepam. She had a penicillin allergy that caused facial swelling.

History of present illness

The patient stated that her left hip pain began several years after the gunshot wound and had slowly worsened over the past 20-30 years. She described the pain as sharp and constant, mainly in the groin and over the greater trochanter. She rated it 8/10 in severity. She also reported clicking and severe stiffness in the hip.

She had tried multiple conservative treatments, including NSAIDs, hydrocodone, shoe lifts, physical therapy, and occasional injections, without lasting relief. She also noticed her left leg gradually shortening over the past 20 years, making daily activities and her work as a customer service representative increasingly difficult.

Physical examination

On examination, the patient walked with a noticeable limp and pelvic tilt. She had a leg length discrepancy of approximately 2.5-3.5 cm, with the left leg being shorter. Examination revealed a healed posterior buttock exit wound scar and significant atrophy of the left thigh muscles. Assessment of the left hip range of motion showed internal rotation of 0°, external rotation of 15-20°, abduction of 10-15°, adduction of 0°, and flexion of 80-90°. Motor strength was 5/5 throughout, distal pulses were palpable, and the limb was neurovascularly intact.

Diagnostic workup

Radiographs

**Figure 1:**
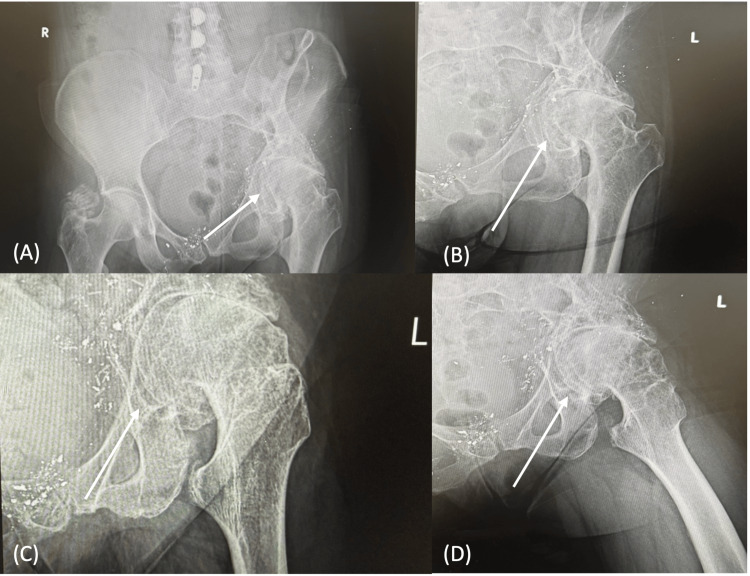
Preoperative hip and pelvis radiographs. (A) Anteroposterior (AP) pelvis radiograph demonstrating severe end-stage left hip osteoarthritis with complete joint space loss, pelvic obliquity, femoral head deformity, and retained metallic fragments from a prior gunshot wound. (B and C) AP view of the left hip showing marked osteophyte formation and femoral head deformity. (D) Frog-leg lateral view of the left hip demonstrating advanced degenerative changes and loss of joint congruency.

**Figure 2:**
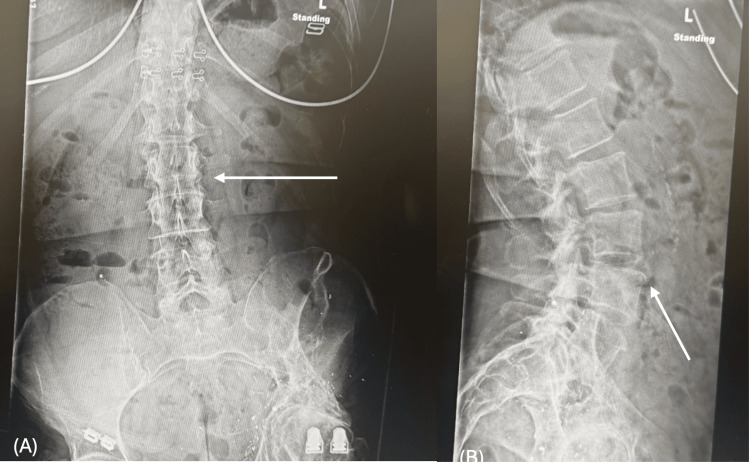
Preoperative lumbar spine radiographs. (A) Anteroposterior view of the lumbar spine showing a gentle compensatory curvature related to pelvic obliquity. (B) Lateral view of the lumbar spine demonstrating mild lower lumbosacral osteoarthritic changes.

Radiographs revealed severe end-stage osteoarthritis, including complete joint space loss and large osteophytes. The deformity of the femoral head, acetabular dysplasia, and retained metallic fragments from the prior injury emphasized the complexity of her condition, while the pelvis remained intact (Figure 1). Lumbar spine X-rays demonstrated mild lower lumbosacral osteoarthritis with compensatory curvature secondary to pelvic obliquity (Figure 2).

Computed Tomography (MAKO Protocol)

**Figure 3:**
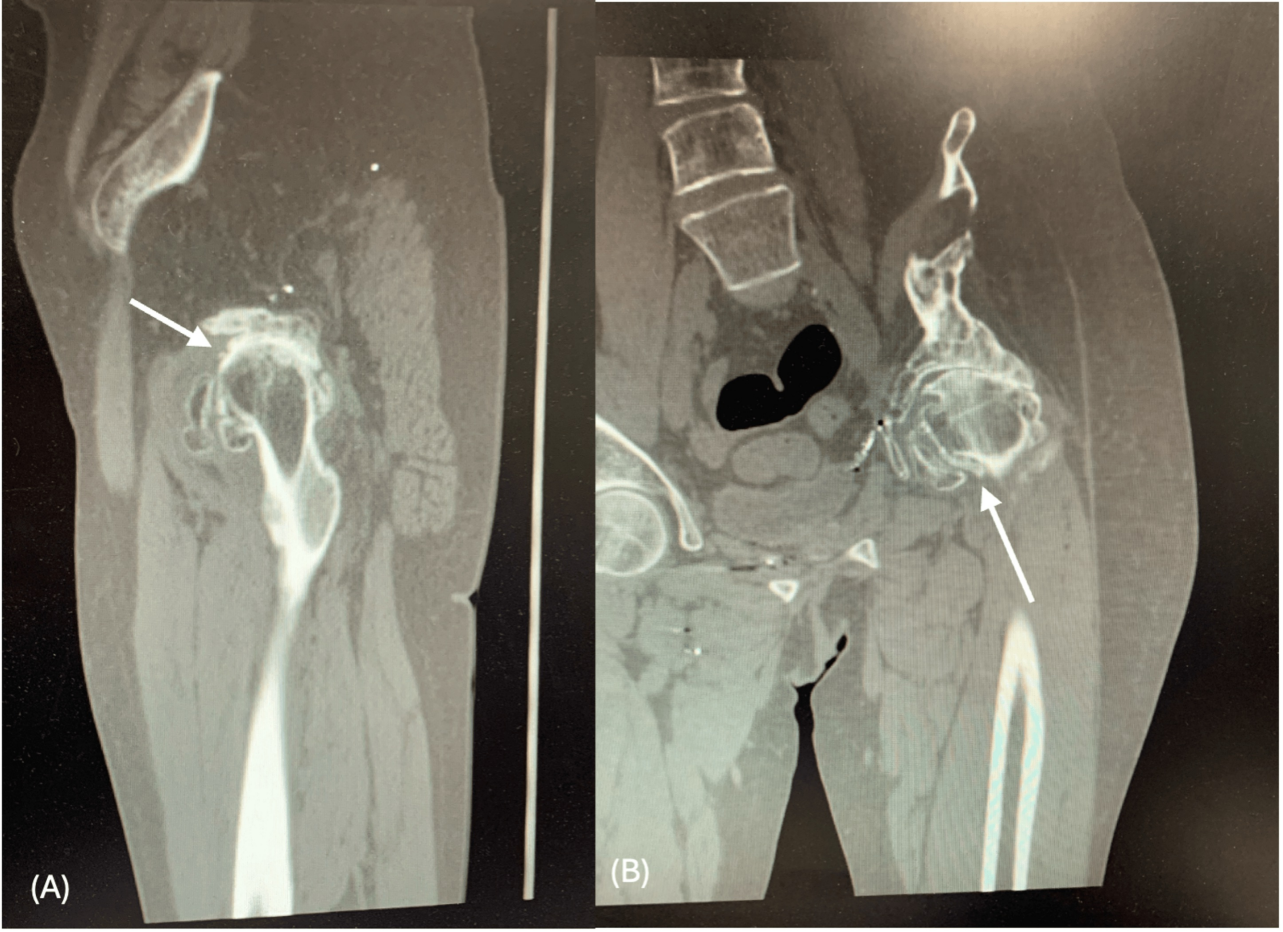
Preoperative computed tomography (MAKO protocol) of the pelvis and left hip. (A) Lateral view demonstrating advanced degenerative changes with osteophyte formation and anterior heterotopic ossification. (B) Anteroposterior view showing chronic left iliac wing fracture deformity with retained metallic fragments and associated pelvic tilt.

CT imaging showed severe left hip osteoarthritis with bone-on-bone contact, diffuse subchondral sclerosis, and large osteophytes measuring up to 12 mm in maximal projection from the native cortical margin, along with a 2.4 cm area of heterotopic ossification along the anterior joint capsule (Figure 3). There was a chronic fracture deformity of the left iliac wing with multiple metallic fragments, superior pelvic tilt, and severe fatty atrophy (≥50% fatty replacement of muscle volume) of the left gluteus medius and minimus. Additional findings included a healed fracture deformity of the right parasymphyseal pubic bone and cortical thickening of the right femur. There were no acute fractures or soft-tissue collections. These findings confirmed that the pelvis was intact and suitable for standard acetabular and femoral components. MRI was not performed because of retained metal fragments.

Assessment

The patient was diagnosed with unilateral post-traumatic osteoarthritis of the left hip with associated leg length discrepancy, pelvic obliquity, heterotopic ossification, and retained metallic fragments due to her prior gunshot wound.

Management

We discussed all treatment options, potential risks, and expected outcomes. Because only partial leg-length correction was likely due to her fixed pelvic obliquity and spinal compensation, she decided to move forward with left THA [4,5].

We selected a direct anterior approach, which offered the best exposure given her prior surgeries and helped protect the surrounding soft tissues. We used preoperative CT templating to plan about 1 cm of leg lengthening and guide implant selection and positioning, minimizing the risk of nerve stretch injury [6,7].

Surgical procedure

On November 21, 2025, the patient underwent direct anterior left THA under spinal anesthesia. Intraoperative steps included removal of a 3 × 3 cm loose body from the anterior femoral neck, femoral neck cut under fluoroscopic guidance, acetabular reaming to 52 mm with medialization to avoid further shortening, placement of a 52 mm acetabular cup with one 6.5 × 30 mm screw and a 0° liner, femoral broaching to size 4, trialing with a +2.5 mm head to achieve approximately 1 cm of leg lengthening, and final implantation with stable reduction. The hip remained stable through a full range of motion, and no intraoperative complications were encountered.

Postoperative course

**Figure 4:**
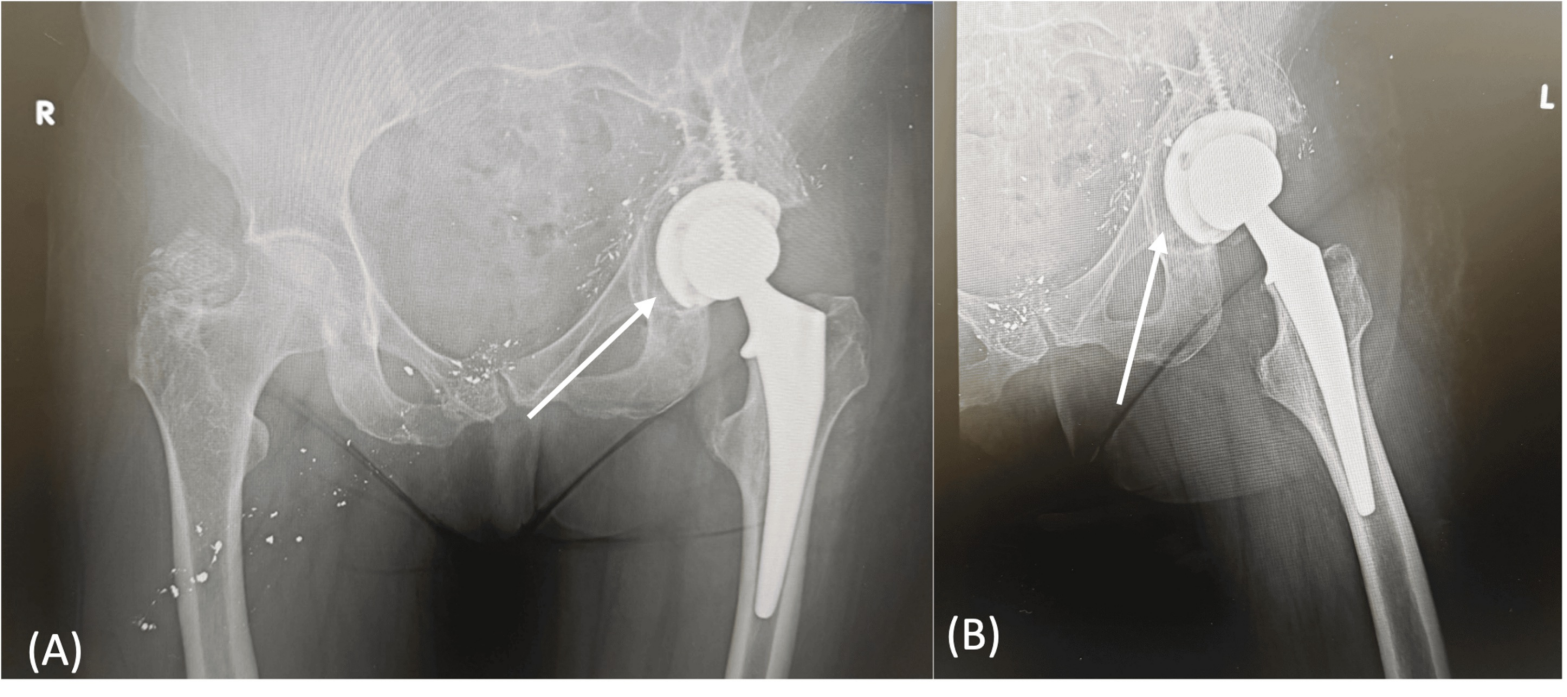
Postoperative hip radiographs. (A and B) Anteroposterior pelvis radiograph demonstrating stable positioning of left THA components without evidence of loosening or subsidence. THA: Total hip arthroplasty

Postoperatively, the patient was allowed to bear weight as tolerated and was started on aspirin 81 mg twice daily for DVT prophylaxis. Radiographs demonstrated proper implant positioning without loosening or subsidence (Figure 4). Two weeks after surgery, she reported daily improvement and ambulated with a cane, and her incision was healing well without signs of infection. Formal physical therapy had not yet started due to scheduling difficulties, but functional gains were observed. She will undergo routine postoperative surveillance at six weeks, three months, six months, and one year, with continued follow-up for a minimum of 1-2 years and periodic long-term evaluation thereafter to monitor implant stability, function, and potential late complications.

## Discussion

This case highlights how a pelvic gunshot wound sustained in adolescence can gradually lead to severe hip osteoarthritis decades later, illustrating the long-term orthopedic consequences [1,2]. Growth arrest, pelvic obliquity, acetabular deformity, heterotopic ossification, and progressive leg length discrepancy all contributed to early-onset end-stage hip osteoarthritis [1,2].

Performing THA in post-traumatic hips is challenging because of distorted anatomy, retained foreign bodies, soft-tissue contractures, gluteal muscle atrophy, and the risk of nerve injury when correcting limb length [3-5]. In this patient, CT imaging was especially important to confirm pelvic continuity, identify heterotopic ossification, and help with surgical planning. MRI could not be used because of retained shrapnel, highlighting the need for alternative imaging options [6,7].

The direct anterior approach allowed the use of fluoroscopy to help with implant positioning and limb length assessment [7]. A planned partial correction of about 1 cm helped balance improved biomechanics with the risk of nerve stretch injury [4,6].

Her early recovery was encouraging: the implants remained stable, and she had already started regaining function by her two-week follow-up. While it’s still too early to know her long-term outcome, our experience suggests that a direct anterior THA can be a promising approach for patients with complex post-traumatic hip pathology, provided that careful planning and cautious lengthening are prioritized [4,5].

## Conclusions

THA can be successfully performed in patients with severe post-traumatic hip osteoarthritis and pelvic deformity many years after an adolescent gunshot wound. Preoperative CT planning, realistic goals for limb-length correction, and careful surgical technique are important for achieving a safe and functional outcome. This case underscores the importance of individualized surgical planning and multidisciplinary collaboration in managing patients with complex post-traumatic hip pathology.
